# Study on Construction of and Pharmacodynamics of Baicalin-Minoxidil Co-Loaded Nanogel

**DOI:** 10.3390/pharmaceutics18101308

**Published:** 2026-10-15

**Authors:** Liping Chen, Enhe Liu, Xiao Liu, Shouxin Yi, Shiting Gao, Xin Luo, Xi Pu, Jiaying Long, Yuxin He

**Affiliations:** 1 School of Food and Bioengineering, Xihua University, Chengdu 610039, China; 2 Key Laboratory of Drug Targeting and Drug Delivery Systems, Ministry of Education, West China School of Pharmacy, Sichuan University, Chengdu 610041, China

**Keywords:** androgenetic alopecia, nanogel, transdermal drug delivery, hair follicle regeneration, Wnt/β-catenin signaling

## Abstract

Androgenetic alopecia (AGA) is a chronic and highly prevalent hair loss disorder for which current topical therapies are limited by undesirable side effects, dose-dependent efficacy, and poor skin penetration. **Objective**: Here, we developed a baicalin-minoxidil co-loaded nanogel (Bai-Min-NGs) to synergistically enhance transdermal drug delivery and promote hair regeneration. **Methods**: The optimized nanogel exhibited favorable colloidal attributes, featuring a nanoscale hydrodynamic diameter of 156 ± 3.62 nm, narrow size distribution (polydispersity index of 0.144 ± 0.011), surface zeta potential of −26.1 ± 0.37 mV, high encapsulation efficiencies (95.86 ± 1.34% for baicalin and 82.16 ± 2.50% for minoxidil), and satisfactory drug loading capacities (1.92 ± 0.08% and 1.64 ± 0.06%, respectively). **Results**: Enhanced cellular internalization through endocytosis further promoted the proliferation and migration of human dermal papilla cells, demonstrating a synergistic biological effect beyond either drug alone. In an androgenetic alopecia mouse model, topical Bai-Min-NGs significantly accelerated hair regrowth, as evidenced by increased hair length, hair weight, hair follicle density, and dermal thickness. Notably, the nanogel achieved superior therapeutic efficacy while maintaining excellent local and systemic biosafety. Mechanistically, Bai-Min-NGs activated the Wnt/β-catenin signaling pathway by upregulating Wnt7b and Wnt10b expression, suppressing TGF-β1, and increasing the protein expression of GSK3β, phosphorylated GSK3β, β-catenin, and VEGF, thereby promoting hair follicle regeneration and angiogenesis. **Conclusions**: Collectively, Bai-Min-NGs provide a promising topical co-delivery nanoplatform integrating superior cutaneous retention with endogenous pathway activation for hair regeneration therapy.

## 1. Introduction

Androgenetic alopecia (AGA) is a pervasive dermatological condition affecting hundreds of millions of people worldwide, characterized by progressive follicular miniaturization and disrupted hair growth cycles [1]. Despite the availability of various therapeutic modalities, minoxidil (Min), the first-line clinical drug, still faces formidable challenges in practical application. On one hand, commercially available formulations typically contain high concentrations of ethanol and propylene glycol, which frequently lead to severe allergic contact dermatitis and scalp dryness upon long-term use. On the other hand, prolonged administration of minoxidil itself is plagued by adverse reactions, including contact dermatitis and facial hypertrichosis [2]. Second, the dense stratum corneum severely restricts the transdermal penetration of Min, resulting in limited drug delivery to hair follicles [3]. Consequently, developing a novel delivery system that enhances skin penetration while reducing systemic side effects is a critical challenge in hair regeneration research. Biocompatible nanocarriers have shown great potential in the fields of tissue engineering and controlled release [4].

Recently, the therapeutic potential of natural products in AGA management has gained significant traction. Baicalin (Bai), a bioactive flavonoid derived from Scutellaria baicalensis, has demonstrated potent anti-inflammatory and antioxidant properties, alongside the ability to promote follicular epithelial cell proliferation [5]. This challenge stems from their incompatible solubilities and distinct release kinetics, which hinder uniform dispersion and coordinated delivery. Moreover, the follicular barrier further restricts penetration, especially for hydrophilic drugs like Minoxidil. Recent advances in nanocarrier engineering—such as amphiphilic polymeric micelles and lipid-polymer hybrid nanoparticles—offered promising solutions by enabling co-encapsulation, stabilizing both actives, and facilitating receptor-mediated follicular targeting [6].

Nanogels, as smart nanocarriers with a three-dimensional network structure, offer a promising opportunity to overcome the aforementioned bottlenecks. Unlike traditional creams or liquid formulations, nanoemulsion-embedded nanogels (NE-nanogels) significantly enhance drug solubility. Furthermore, their inherent structural flexibility allows them to deform and penetrate through follicular orifices, thereby bypassing the stratum corneum to act directly on dermal papilla cells (DPCs) [7,8]. Additionally, the prolonged adhesion provided by the gel matrix extends the residence time at the target site, creating a “drug reservoir” for sustained release [9]. This mechanism not only bolsters therapeutic efficacy but also reduces the required dosing frequency [10].

In this study, a dual-drug loaded nanogel system (Bai-Min-NGs) was designed and synthesized to co-deliver Bai and Min. We systematically evaluated its rheological properties, skin permeation behavior, and biosafety. Furthermore, its superior hair regrowth capacity was validated using both C57BL/6 mouse models and DPCs. Significantly, the underlying therapeutic mechanism was elucidated at the molecular level; Bai-Min-NGs were found to activate the Wnt/β-catenin signaling pathway, leading to the marked upregulation of the core regulatory factor β-catenin and the subsequent promotion of DPC proliferation and migration [11]. These findings not only offer a highly efficient, low-toxicity pharmaceutical strategy for the clinical management of AGA but also advance the development of nanogel-based topical drug delivery systems for hair regeneration.

## 2. Materials and Methods

### 2.1. Materials

Cell culture consumables, including 6-well plates (Corning, Corning, NY, USA), 100 mm culture dishes, and 15 and 50 mL centrifuge tubes (Labselect, Shanghai, China), were utilized. Trypsin-EDTA solution was purchased from Yuanpei Biotechnology (Shanghai, China). For fluorescence staining, LysoTracker Red DND-99 (Invitrogen, Carlsbad, CA, USA), Coumarin 6 (Macklin, Shanghai, China), and 4′,6-diamidino-2-phenylindole (DAPI) (Solarbio, Beijing, China) were employed. Dimethyl sulfoxide (DMSO) (Bomei, Shanghai, China), ethanol (Xilong Scientific, Guangzhou, China), 4% paraformaldehyde (Biosharp, Hefei, China), and phosphate-buffered saline (PBS) (Procell Life Science & Technology, Wuhan, China) were used as general reagents. Six-week-old C57BL/6 mice were obtained from Speifur (Beijing) Biotechnology Co., Ltd., Beijing, China (License No. SCXK (Beijing) 2024-0001). New Zealand White rabbits were purchased from Chengdu Dashuo Experimental Animal Co., Ltd., Chengdu, China (SCXK (Chuan) 2022-0039). All animals were housed in a specific-pathogen-free (SPF) facility under a 12 h light/dark cycle at 25 ± 1 °C. After a one-week acclimatization period, the animals were subjected to experimental procedures. (IACUC-SWLAB-2025-10-11-05).

### 2.2. Cell Lines and Cells

The human primary dermal papilla cell culture system (Cyagen Biosciences, Shanghai, China; Catalog No. HUM-iCell-s005) was used as the cell source for all in vitro experiments. Cells were cultured in the medium supplemented with 10% fetal bovine serum (FBS) and 1% penicillin-streptomycin at 37 °C in a humidified atmosphere containing 5% CO_2_. The culture medium was refreshed every 2–3 days, and cells were passaged upon reaching 80–90% confluence using Trypsin-EDTA solution. All experiments were performed with cells at passages 3–6 to ensure phenotypic stability.

### 2.3. Preparation of Bai-Min-NEs and Bai-Min-NGs

#### 2.3.1. Preparation of Bai-Min-NEs

The aqueous phase (containing Tween-80 and Span-80 in water) and the lipid phase (containing Bai, Min, stearic acid, and oleic acid in oil) were separately heated to 80 °C and then mixed. The mixture was homogenized at 10,000 rpm for 2 min using a high-speed homogenizer (T18; Shanghai Binjiang Scientific Instrument Co., Ltd., Shanghai, China) and then processed through a microfluidizer (Noze Fluid Technology Co., Ltd., Shanghai, China) for four cycles at a pressure of 10,000 psi to yield Bai-Min-NEs. After cooling to 25 °C, the Bai-Min-NEs was characterized [12].

#### 2.3.2. Preparation of Bai-Min-NGs

Carbomer 934, Poloxamer 407, and Poloxamer 188 were slowly dispersed in deionized water, followed by the addition of the nanoemulsion at the target concentration. The mixture was gently stirred for 2 h, neutralized with triethanolamine (TEA) to trigger gelation, and then left overnight for complete crosslinking and bubble elimination, eventually yielding the Bai-Min-NGs.

### 2.4. Of Bai-Min-NEs and Bai-Min-NGs

#### 2.4.1. Morphological Characterization via TEM

The morphology of Bai-Min-NGs was characterized using transmission electron microscopy (TEM). Briefly, a copper grid was immersed in the diluted sample solution, placed on filter paper, and dried at 25 °C. For negative staining, a drop of phosphotungstic acid (2%, *w/v*) was applied to the grid. The samples were then observed under a TEM (Talos F200X; Thermo Fisher Scientific, Waltham, MA, USA).

#### 2.4.2. Physicochemical Properties and Encapsulation Efficiency

The particle size, polydispersity index (PDI), and zeta potential of Bai-Min-NEs were determined by a Zetasizer Nano ZS90 (Malvern Instruments, Worcestershire, UK). Prior to analysis, all samples were diluted to an appropriate concentration with ultrapure water. The temperature was maintained at 25 °C, and all measurements were performed in triplicate [13,14].

To determine the total drug content, Bai-Min-NEs were dissolved in methanol and subjected to ultrasonication for 30 min. The free drug content was obtained by dissolving the nanoemulsion in methanol followed by direct filtration. The drug concentrations were subsequently quantified using high-performance liquid chromatography (HPLC) [15]:

$$\mathrm{EE}\;(\%)\;=\;\frac{\mathrm{total}\;\mathrm{amout}\;\mathrm{of}\;\mathrm{loaded}\;\mathrm{NEs}-\mathrm{amount}\;\mathrm{of}\;\mathrm{free}\;\mathrm{NEs}}{\mathrm{total}\;\mathrm{amount}\;\mathrm{of}\;\mathrm{loaded}\;\mathrm{NEs}}\times 100\%$$

#### 2.4.3. Rheological Properties and Evaluation

All rheological characterizations of Bai-Min-NGs were performed using an MCR302 rheometer (Chengdu) equipped with a PP25 parallel plate geometry. The experimental temperature was maintained at 25 ± 1 °C with a fixed gap of 1000 µm [16]. To determine the linear viscoelastic region (LVR), strain-controlled amplitude sweeps were conducted at a constant frequency of 10 Hz over a shear strain range of 0.01–1%. This analysis also served to evaluate the strain dependence of the storage modulus (G′) and loss modulus (G″) [17,18].

Frequency sweeps were subsequently performed at a constant shear strain of 0.1% across a range of 100–1 rad/s to assess the frequency-responsive characteristics of G′ and G″. Furthermore, the flow behavior was investigated by recording the viscosity and shear stress as the shear rate was increased from 0.01 to 100 s^−1^ over a 100 s interval to generate the steady-state viscosity curves.

#### 2.4.4. FT-IR Spectroscopic Analysis

The chemical structures of the lyophilized Bai-Min-NGs were characterized using Fourier-transform infrared spectroscopy (FT-IR). Briefly, the samples were mixed with potassium bromide (KBr) at an appropriate ratio, ground into a fine powder, and compressed into translucent pellets. Spectra were recorded in the range of 4000–650 cm^−1^ using a Nicolet Avatar 330 FT-IR spectrometer (Thermo Nicolet Corp., Madison, WI, USA).

#### 2.4.5. Differential Scanning Calorimetry

The thermal behavior of the lyophilized Bai-Min-NGs was analyzed via differential scanning calorimetry (DSC) using a DZ-DSC300C differential scanning calorimeter (Nanjing, China). Accurately weighed samples were sealed in flat-bottomed aluminum pans and heated from 30 °C to 200 °C at a constant scanning rate of 10 °C/min under a continuous nitrogen purge (60 mL/min). The resulting thermograms were recorded to identify thermal transition characteristics.

### 2.5. In Vitro Drug Release Studies

The in vitro release profiles of the nanoemulsion and nanogel were evaluated using Franz diffusion cells. Briefly, 1 mL of the formulation was loaded into the donor compartment, which was separated from the receptor compartment by excised mouse skin. The receptor compartment was filled with isothermal physiological saline. At predetermined time intervals (0, 0.25, 0.5, 1, 2, 3, 4, 5, 6, 8, 10, 12, and 24 h), a 1 mL aliquot was withdrawn from the receptor compartment for analysis and immediately replaced with an equal volume of fresh saline to maintain sink conditions [19].

To elucidate the release mechanism, the in vitro release profiles were fitted into four mathematical kinetic models: Zero-order (Q = k_0_·t + a), First-order (ln(100−Q) = −k_1_·t + a), Higuchi (Q = k_H_·t^1/2^ + a), and Korsmeyer–Peppas (ln(Mt/M∞) = nlnt + lnk_KP_, for Mt/M∞ ≤ 0.60). The correlation coefficient (R^2^) and release exponent (*n*) were evaluated.

### 2.6. Ex Vivo Skin Permeation Experiments

To evaluate the skin penetration and deep-tissue delivery of Bai-Min-NGs, we conducted a fluorescence distribution study using Coumarin-6 (C6) as a fluorescent probe. The C6-loaded nanosuspensions and nanogels were topically applied to the depilated dorsal skin of mice. At predetermined time intervals, skin samples were harvested from the application site, immediately snap-frozen in liquid nitrogen, and embedded in optimal cutting temperature (OCT) compound. Transverse sections (10 μm thick) were prepared using a cryostat microtome (Leica-2016, Leica Microsystems, Wetzlar, Germany) and subsequently visualized via fluorescence microscopy (Nikon Eclipse Ci, Tokyo, Japan) to characterize the depth and intensity of skin permeation.

### 2.7. In Vitro Cell Experiments

#### 2.7.1. Cell Culture

Cells were rapidly thawed in a 37 °C water bath, diluted with three volumes of pre-warmed medium, and centrifuged at 250 *g* for 5 min. The resulting pellet was resuspended and incubated in a humidified CO_2_ incubator. Upon reaching confluence, cells were detached using 0.25% trypsin. Once the cells rounded up and detached from the flask wall, the reaction was neutralized with three volumes of complete medium. Finally, cells were subcultured at a split ratio of 1:3.

#### 2.7.2. Cell Proliferation Assay

DPCs were seeded in 96-well plates at a density of 5 × 10^4^ cells/mL (100 μL per well). Peripheral wells were filled with PBS to minimize edge effects. After 24 h incubation, cells were assigned to the model, control, and blank groups, as well as treatment groups receiving baicalin–minoxidil gel, suspension, baicalin gel, or minoxidil gel. Drug concentrations ranged from 0.1 to 10 μg/mL, with four replicates per group. Following 24 h of treatment, 110 μL of CCK-8 working solution (diluted 1:10) was added to each well and incubated for 2 h. The optical density (OD) was measured at 450 nm to evaluate cell viability.

#### 2.7.3. Wound-Healing Assay

DPCs were seeded in 6-well plates at a density of 3 × 10^5^ cells per well. Upon reaching a confluent monolayer, a linear scratch was created using a 200 μL pipette tip held perpendicular to the cell layer. After washing twice with PBS, cells were cultured in serum-free medium containing different concentrations of drugs (0.5, 2, and 5 μg/mL). The experimental groups included a control group, blank gel group, minoxidil group, baicalin group, as well as the corresponding single-drug gels and combined gel formulation. Images were captured at 0 h and 24 h under the same field of view (50× magnification), and the wound closure rate was calculated to evaluate cell migratory capacity.

#### 2.7.4. Flow Cytometry Analysis

DPCs were seeded in 6-well plates at a density of 3 × 10^5^ cells per well. After cell attachment, they were divided into a control group, a free C6 group, and a C6-labeled nanogel group (C6, 2 μg/mL; drug loading, 2%), with three biological replicates per group. Following 2 h incubation, cells were harvested, dissociated with EDTA-free trypsin, washed with PBS, and resuspended. Cellular uptake was quantitatively analyzed by flow cytometry.

#### 2.7.5. Cellular Uptake and Fluorescent Imaging

After treatment, cells were incubated with 50 μM LysoTracker Red DND-99 for 90 min to label lysosomes, followed by fixation with 4% paraformaldehyde for 15 min. Nuclei were counterstained with DAPI for 5 min. Intracellular fluorescence distribution was observed and recorded using an inverted fluorescence microscope.

### 2.8. In Vivo Therapeutic Efficacy on AGA Mice Model

To evaluate the efficacy of the synthesized nanogels on hair regeneration, an androgenetic alopecia (AGA) model was established using male C57BL/6 mice (6 weeks old, 18–20 g, *n* = 42) [20]. Following a one-week period of acclimatization, the dorsal hair of the mice was removed using an electric shaver and a topical depilatory cream.

The animals were randomly assigned to seven experimental groups (*n* = 6 per group): (A) Model (untreated AGA); (B) Control (Commercial 2% minoxidil tincture); (C) Blank (normal mice without AGA induction); (D) Bai-Min-NGs (experimental nanogels); (E) Baicalin-Minoxidil Suspension; (F) Baicalin Gel; (G) Minoxidil Gel.

After physical depilation, the Blank group was allowed to undergo natural hair cycles. To induce AGA in the remaining six groups, 0.2% (*w/v*) testosterone ethanol solution (TES) (dissolved in 50% ethanol) was applied topically to the denuded dorsal area daily [20].

To induce AGA, 100 μL of 0.2% (*w/v*) testosterone ethanol solution (TES, in 50% ethanol) was applied topically to the denuded area daily at 9:00 AM. Treatments were administered topically at 2:00 PM daily (2 mL gel or 100 μL liquid per application, delivering an equivalent dose of X mg Minoxidil and Y mg Baicalin per mouse) for 21 consecutive days.

### 2.9. Mechanistic Studies on wnt/β-Catenin Signaling Pathway

#### 2.9.1. RNA Extraction and RT-qPCR Assay

Total RNA was extracted from mouse skin tissue using the TRIzol method, followed by reverse transcription to synthesize cDNA. Quantitative PCR (qPCR) was performed using SYBR Green dye on a real-time PCR system. Relative gene expression levels were calculated using the 2- Ct method.

#### 2.9.2. Protein Extraction and Western Blot Analysis

Mouse skin tissues were homogenized in RIPA lysis buffer supplemented with protease inhibitors at a 1:10 (*w/v*) ratio. After incubation on ice for 30 min, the lysates were centrifuged, and the resulting supernatants were collected and denatured. Proteins were separated by SDS-PAGE and transferred onto PVDF membranes. Following a blocking step, the membranes were incubated sequentially with specific primary antibodies and HRP-conjugated secondary antibodies. Protein bands were visualized using enhanced chemiluminescence (ECL), and their densitometric values were quantified using image analysis software. The relative expression levels of the target proteins were normalized to the internal control.

### 2.10. Safety Evaluation

#### 2.10.1. Repeated Skin Irritation Test in Rabbits

Repeated skin irritation tests were performed using New Zealand white rabbits. A total of 1 animals were included, and a self-controlled design with left and right dorsal sites was adopted: Bai-Min-NGs were applied topically to the left dorsal skin, while an equal volume of normal saline was applied to the right dorsal skin. The intact dorsal skin of rabbits was depilated and allowed to recover for 24 h. The administration was performed three times daily for 7 consecutive days. Residual formulations were gently removed with warm water before each dosing. At 1 h after each application, skin reactions including erythema, eschar and edema were scored in accordance with the Technical Specification for Cosmetic Safety (2015 Edition). The mean cumulative score was calculated based on skin reaction scores at each observation time point to determine the irritation grade.

#### 2.10.2. Systemic Histopathological and Biosafety Assessment

Histopathological assessment of mouse skin and major organs (heart, liver, spleen, lung, and kidney) was performed using hematoxylin and eosin (H&E) staining to evaluate the biosafety of the nanocarrier system. The integrity of skin architecture, morphological changes in the epidermis and dermis, and the presence of any abnormalities were examined. In addition, cellular morphology in the heart, liver, spleen, lung, and kidney was evaluated to identify any pathological alterations or tissue damage. The safety of Bai-Min-NGs was determined based on the observed histological features.

### 2.11. Statistical Analysis

Statistical analysis was performed using GraphPad Prism 9 (GraphPad Software, San Diego, CA, USA). All data are presented as mean ± SEM [21]. Differences among groups were evaluated by one-way analysis of variance (one-way ANOVA) based on data obtained from three independent experiments. Statistical significance was defined as follows: ns, not significant; * *p* < 0.05, ** *p* < 0.01, *** *p* < 0.001, and **** *p* < 0.0001 (vs. model group); # *p* < 0.05 and ## *p* < 0.01 (vs. normal group).

Additionally, A post hoc power analysis was performed using G*Power (version 3.1.9.7). Based on the primary endpoints (hair length and follicle density), the effect size (Cohen’s f) exceeded 1.40, and the statistical power (1−β) was greater than 0.99 at α = 0.05 with *n* = 6 per group, confirming adequate sample size.

## 3. Results

### 3.1. Characterization of Bai-Min-NEs and Bai-Min-NGs

**Figure 1:**
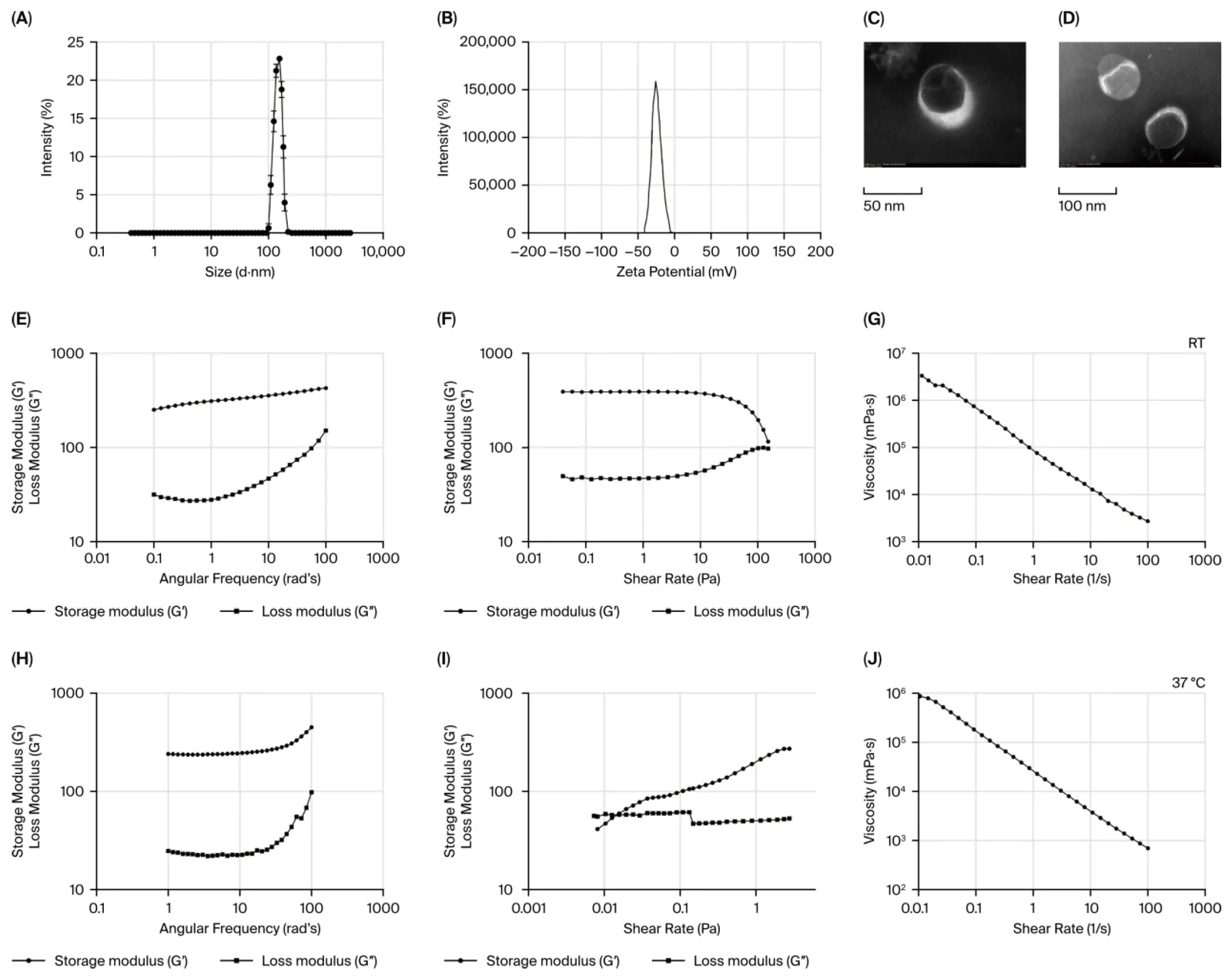
(**A**) Scatter plots of particle size distribution and (**B**) the zeta potential of Bai-Min-NEs. (**C**) TEM images of Bai-Min-NGs at 50 nm and (**D**) at. 100 nm. Rheology images of Bai-Min-NGs. (**E**) amplitude tests at 25 °C. (**F**) Frequency sweep test at 25 °C and (**G**) viscosity curve at 25 °C. (**H**) amplitude tests at 37 °C. (**I**) Frequency sweep test at 37 °C and (**J**) viscosity curve at 37 °C.

The physicochemical properties of Bai-Min-NEs were characterized utilizing a Malvern Zetasizer NANO ZS (Malvern Panalytical, Malvern, UK). The measurement results demonstrated that Bai-Min-NEs possessed an average hydrodynamic diameter of 156 ± 3.62 nm, and the size distribution curve exhibited a symmetrical unimodal distribution (Figure 1A), indicating a homogeneous dispersion of the system [22]. The PDI of Bai-Min-NEs was as low as 0.144 ± 0.011, suggesting that the nanoparticles possessed excellent monodispersity [23]. Furthermore, the surface zeta potential of Bai-Min-NEs was −26.1 ± 0.37 mV (Figure 1B). Its absolute value was greater than 20 mV, ensuring that the nanoemulsion maintained sufficient electrostatic repulsion and favorable physical stability [24]. Regarding the drug-loading performance, the EE of Bai-Min-NEs for baicalin and minoxidil reached 95.863 ±1.338% and 82.158 ± 2.495%, respectively [25], which is beneficial for the subsequent drug delivery into the deep layers of the skin.

To prolong the retention time of the drugs in the local skin, the aforementioned high-drug-loading nanoemulsion was dispersed into a cross-linked matrix to prepare Bai-Min-NGs. The microscopic morphology was observed via TEM. As shown in Figure 1C,D, the TEM micrographs revealed that the Bai-Min-NGs particles exhibited a regular spherical structure with a smooth surface and no obvious aggregation, and the microscopic particle size was approximately 150 nm. This result was highly consistent with the hydrodynamic diameter of the liquid nanoemulsion measured by DLS, indicating that the nanoemulsion maintained its original fine nanostructure and good regularity after being encapsulated and cross-linked into the gel network. Such excellent morphological characteristics are conducive to increasing the contact time with skin tissues, thereby improving bioavailability [26,27].

Rheological testing further evaluated the mechanical structure and spreadability of the nanogel. In the amplitude sweep test (Figure 1E), Bai-Min-NGs exhibited a clear linear viscoelastic region (LVR), within which the storage modulus (G′) was significantly higher than the loss modulus (G″), indicating that the system possessed a typical elastic solid behavior and a stable gel network under static conditions [28]. Subsequently, the angular frequency sweep test (Figure 1F) showed that under a linear strain value of 0.1%, G′ remained relatively constant and was consistently higher than G″ with increasing angular frequency, which strongly proved that the prepared system was in a stable three-dimensional elastic gel state [28,29]. In addition, the apparent viscosity of the gel decreased sharply with the increase in shear rate (Figure 1G), exhibiting significant shear-thinning behavior. This characteristic allows the formulation to undergo a reduction in viscosity when subjected to local shear forces such as rubbing and friction during topical application, making it easy to spread smoothly on irregular scalp surfaces. Upon the cessation of rubbing (disappearance of the shear force), the gel can rapidly recover its high viscosity to remain firmly localized in the administration area, thereby enhancing clinical applicability and therapeutic efficacy [30,31].

**Figure 2:**
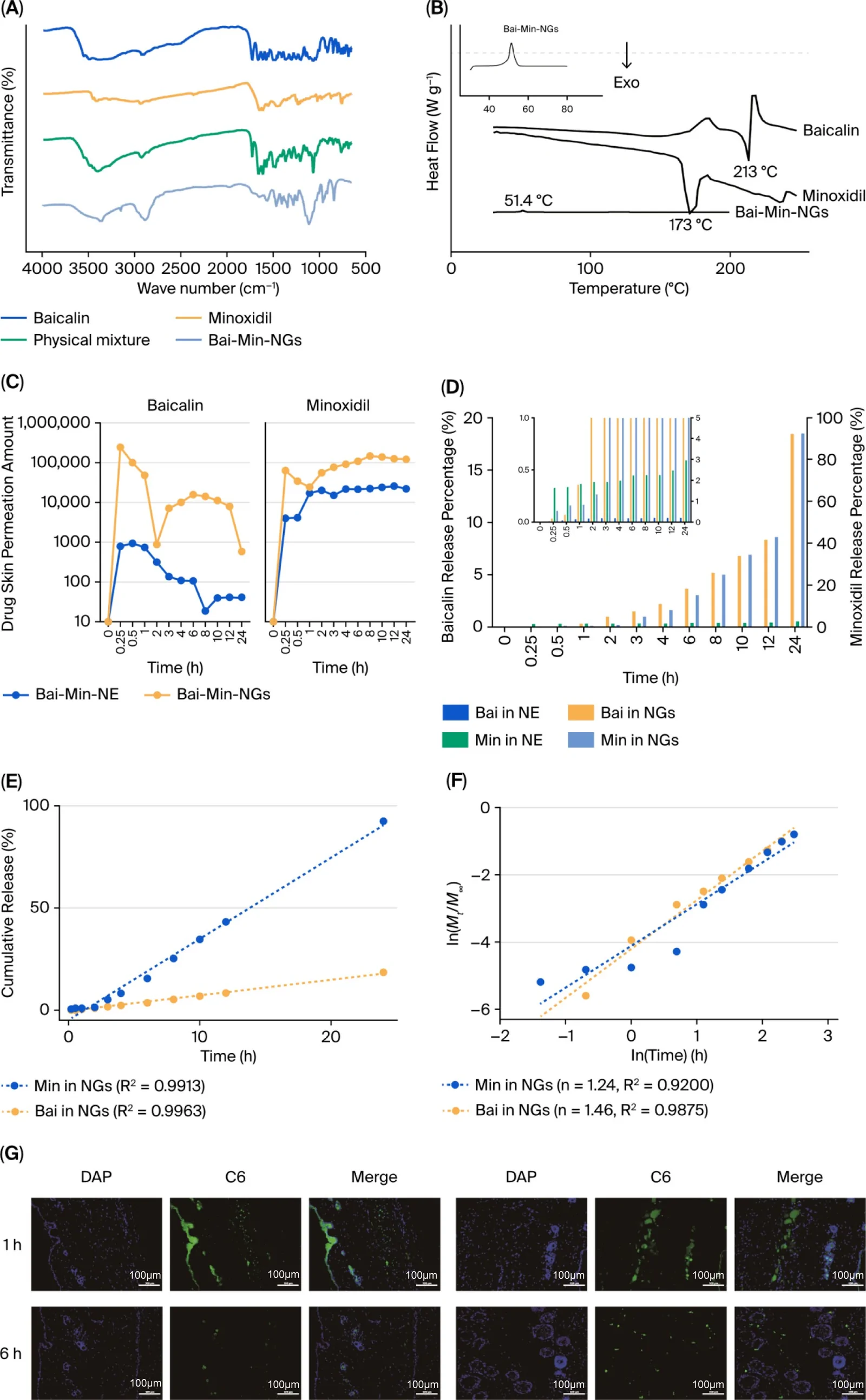
(**A**) FTIR spectra of Baicalin, Minoxidil, Physical Mixture and Bai-Min-NGs. (**B**) DSC analysis of raw drugs and Bai-Min-NGs formulation. (**C**) Release amount curve of Bai-Min- NEs and Bai-Min-NGs. (**D**) Drug release percentage curve of Bai-Min-NEs and Bai- Min-NGs. (**E**) Zero-order kinetics. (**F**) Korsmeyer-Peppas Model fitting. (**G**) Transdermal penetration of Bai-Min-Sus and Bai-Min-NGs.

The intermolecular interactions among the components during the gelation and encapsulation processes were investigated via FT-IR. As shown in Figure 2A, the crude baicalin spectrum exhibited representative characteristic absorption peaks at 3490 cm^−1^, 3390 cm^−1^, 1661 cm^−1^ and 1609 cm^−1^, corresponding to intermolecular hydroxyl groups, free hydroxyl groups, C=C double bonds, and benzene ring C-H stretching vibrations, respectively. The spectrum of minoxidil showed characteristic absorption peaks at 3411 cm^−1^ (-NH_2_), 1644 cm^−1^ (C=C) and 1555 cm^−1^ (C=N). In the physical mixture, the characteristic peaks of both drugs were well preserved. However, in the FT-IR spectrum of the finally formed nanogel (Bai-Min-NGs), the intensities of the characteristic absorption peaks of baicalin and minoxidil were significantly weakened or partially shielded, indicating that the two drugs were successfully embedded inside the carrier [32]. Meanwhile, no new covalent bond peaks were observed in the spectrum, confirming that the drugs and carrier materials interacted primarily through physical entrapment or non-covalent interactions during preparation without undergoing adverse chemical reactions, thereby ensuring the chemical stability of the original drugs [24].

Finally, DSC was employed to analyze the thermal transition behavior of the gel and the physical state of the drugs (Figure 2B). The thermogram results showed that Bai-Min-NGs exhibited a sharp endothermic peak at 51.39 °C, corresponding to the matrix crystalline melting of the system [23,24]. Meanwhile, due to the encapsulation and dispersion effects of the matrix, the original crystalline melting peaks of the pure drugs disappeared, confirming that the drugs had successfully transformed from a crystalline state to an amorphous state and were highly dispersed within the nanogel system. This physical state was highly advantageous for drug release and absorption in skin tissues.

### 3.2. In Vitro Release and Transdermal Penetration Performance

Figure 2C illustrated the in vitro release kinetics of Bai-Min-NEs and Bai-Min-NGs. Over a 24 h period, the cumulative release of baicalin from the Bai-Min-NGs group consistently outperformed that of the NEs group. In contrast, while minoxidil exhibited a slightly faster initial burst in the Bai-Min-NEs group, its release rate remained negligible at less than 1% within the first 2 h. Conversely, the Bai-Min-NGs group demonstrated a superior release profile starting from the 3 h mark, achieving a final cumulative release of approximately 90% and exhibiting favorable sustained-release characteristics [29].

To further investigate the release mechanism under simulated cutaneous microenvironments, four classical kinetic models were applied to fit the cumulative release profiles of Bai-Min-NGs (Table 1). Remarkably, both minoxidil and baicalin exhibited superior linearity with the zero-order kinetic model (R^2^ = 0.9913 and 0.9963, respectively), indicating an ideal, constant sustained-release behavior without burst release (Figure 2E). Furthermore, Korsmeyer–Peppas fitting yielded release exponents (*n*) of 1.2429 (R^2^ = 0.9200) for minoxidil and 1.4553 (R^2^ = 0.9875) for baicalin (Figure 2F). In polymeric drug delivery systems, *n* > 0.89 is characteristic of Super Case-II transport, demonstrating that drug release is predominantly governed by hydrogel network relaxation and matrix swelling rather than concentration-driven Fickian diffusion. This swelling-controlled mechanism effectively rationalizes the steady and extended delivery of the dual actives within the follicular niche.

The in vitro skin permeation results in Figure 2C further confirmed that the cumulative permeation of both drugs in the Bai-Min-NGs group was significantly higher than that in the Bai-Min-NEs group. Specifically, the Bai-Min-NGs formulation achieved a rapid initial burst release of baicalin and ensured a sustained, high-level permeation of minoxidil within 24 h. These findings demonstrated that the nanogel vehicle not only effectively overcame the skin barrier but also optimized the spatiotemporal distribution of the drugs, establishing a robust foundation for localized synergistic therapy.

### 3.3. Effects of Formulations on DPCs Proliferation and Migration

#### 3.3.1. Regulation of DPC Viability

**Figure 3:**
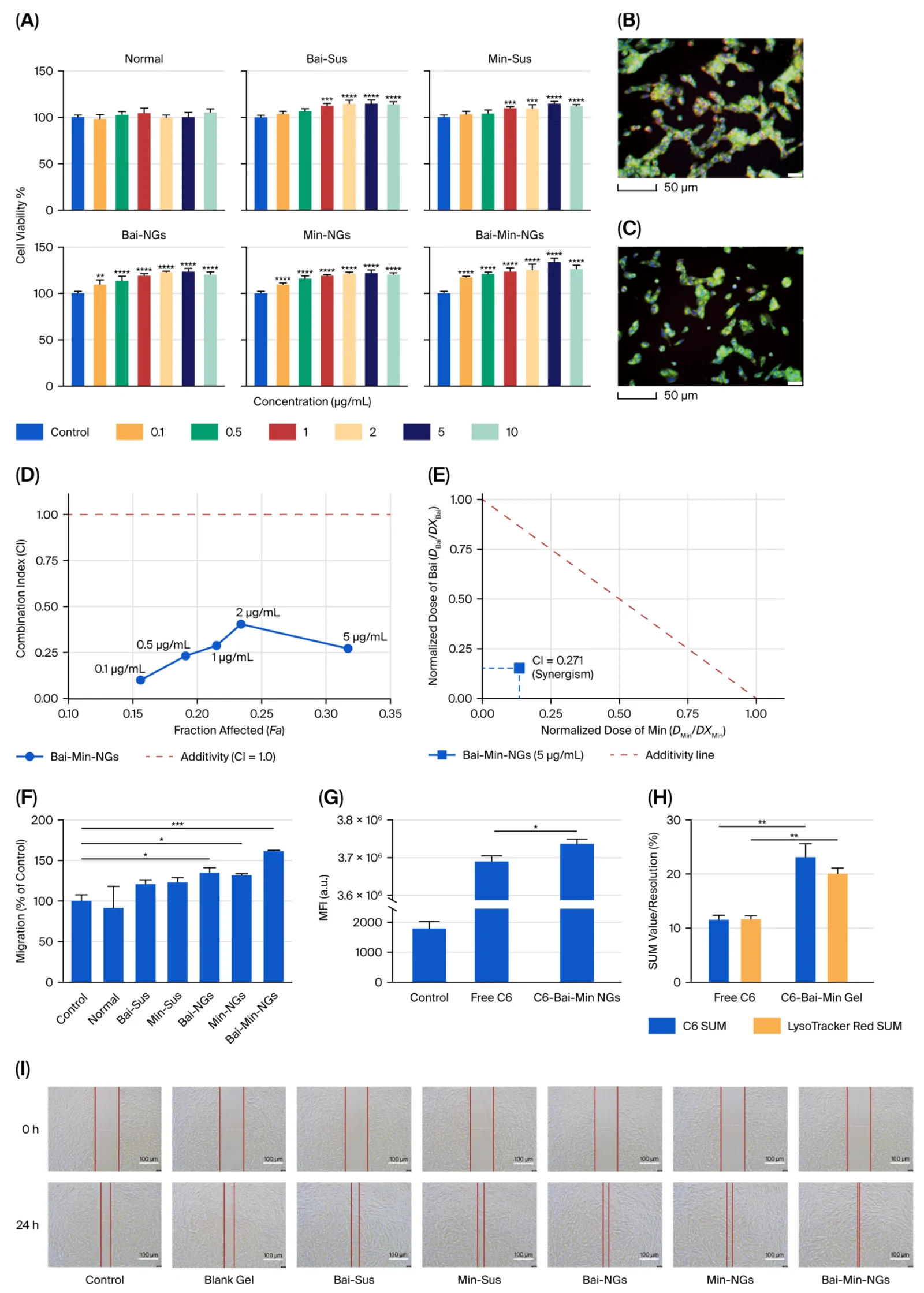
Effects of different treatments on the migration of dermal papilla cells DPCs. (**A**) Representative images of the scratch assay for each treatment group at 0 and 24 h. (**B**,**C**) Enlarged views of the scratch closure in the Bai-Min-NGs group compared to the control group. (**D**) Fa-CI plot constructed based on the Chou-Talalay median-effect principle. (**E**) Normalized isobologram at the optimal effective concentration (5 μg/mL). (**F**) Quantitative analysis of the relative migration rate of DPCs after 24 h of treatment. (**G**) Mean fluorescence intensity (MFl) of C6 uptake in DPCs determined by flow cytometry. (**H**) Semi-quantitative analysis of C6 and LysoTracker Red fluorescence intensity (SUM value/resolution) based on confocal imaging. (**I**) Representative confocal images showing the intracellular distribution and lysosomal colocalization of C6. (* *p* < 0.05, ** *p* < 0.01, *** *p* < 0.001, and **** *p* < 0.0001 (vs. model group)).

The effects of various treatment groups on the viability of DPCs were evaluated using the CCK-8 assay. Bai exhibited a distinct dose-dependent pro-proliferative effect in both the Sus and NGs groups. At a concentration of 5 μg/mL, the Bai-loaded nanogel (Bai-NGs) enhanced cell viability more effectively than its solution counterpart (Figure 3A). Min, serving as the positive control, demonstrated a similar trend, with the nanogel formulation outperforming the solution group in promoting proliferation.

To further quantitatively evaluate the pharmacological interaction between the two actives, the dose–response data across 0.1–5.0 μg/mL were analyzed based on the Chou–Talalay median-effect principle using CompuSyn software (Version 3.0.1, ComboSyn, Inc., Paramus, NJ, USA) (Figure 3D). Compared with each monotherapy, Bai-Min-NGs exhibited superior pro-proliferative activity on DPCs. The combination index (CI) values across all tested concentrations remained well below 1.0 (ranging from 0.100 to 0.405), reaching 0.271 at the optimal concentration of 5.0 μg/mL (Figure 3E). Furthermore, the combination data point in the normalized isobologram was located distinctly below the theoretical additivity line, confirming a robust synergistic effect rather than simple additive interaction. Consistently, the dose-reduction index (DRI) values for minoxidil and baicalin reached 8.8 and 6.4, respectively, indicating that co-encapsulation markedly amplifies the pro-proliferative bioactivity while substantially reducing the required effective therapeutic dose of each individual drug.

#### 3.3.2. Promotion of DPC Migration (Wound Healing)

Quantitative analysis of the wound healing assay (Figure 3F) revealed that the blank hydrogel had no significant effect on the migration of DPCs at the tested concentrations. In contrast, the nanogel formulations (Bai-NGs and Min-NGs) both exhibited significant pro-migratory effects. Notably, the composite nanogel group (Bai-Min-NGs) demonstrated the most potent enhancement of cell migration.

Representative micrographs of the scratch assay were consistent with the quantitative findings. At 24 h post-treatment, the Bai-Min-NGs group displayed the narrowest scratch width and the highest closure rate, further confirming its superior capacity to accelerate DPC migration.

#### 3.3.3. Cellular Uptake and Intracellular Distribution

Flow cytometry analysis revealed that both the free C6 group and the C6-Bai-Min-NGs group exhibited robust fluorescence signals compared to the blank control, confirming successful cellular internalization of C6. Quantitative assessment demonstrated that the mean fluorescence intensity (MFI) of the C6-Bai-Min-NGs group was significantly higher than that of the free C6 group (Figure 3G). These findings verify that the nanogel vehicle effectively facilitates cellular drug uptake and enhances intracellular accumulation levels.

Consistent with the aforementioned trends, CLSM imaging (Figure 3B,C) revealed diffuse and intense green fluorescence within the cytoplasm of the C6-Bai-Min-NGs group, whereas the free C6 group exhibited relatively weak and punctate fluorescence signals. Semi-quantitative analysis further confirmed that the fluorescence intensity of C6 in the nanogel group was significantly superior to that of the free drug group (Figure 3H).

Notably, a significant enhancement of red fluorescence from lysosomes was observed in the nanogel group, accompanied by pronounced intracellular green-red colocalization (Figure 3B,C). Semi-quantitative fluorescence analysis (Figure 3H) and flow cytometry (Figure 3G) consistently demonstrated that the nanogel formulation markedly improved intracellular drug accumulation compared with the free probe (*p* < 0.05). These findings indicate that Bai-Min-NGs efficiently facilitate cellular internalization via the endo-lysosomal trafficking pathway, significantly altering intracellular drug distribution and establishing an intracellular reservoir for sustained biological activity.

### 3.4. Therapeutic Efficacy of Bai-Min-NGs in AGA Mice

**Figure 4:**
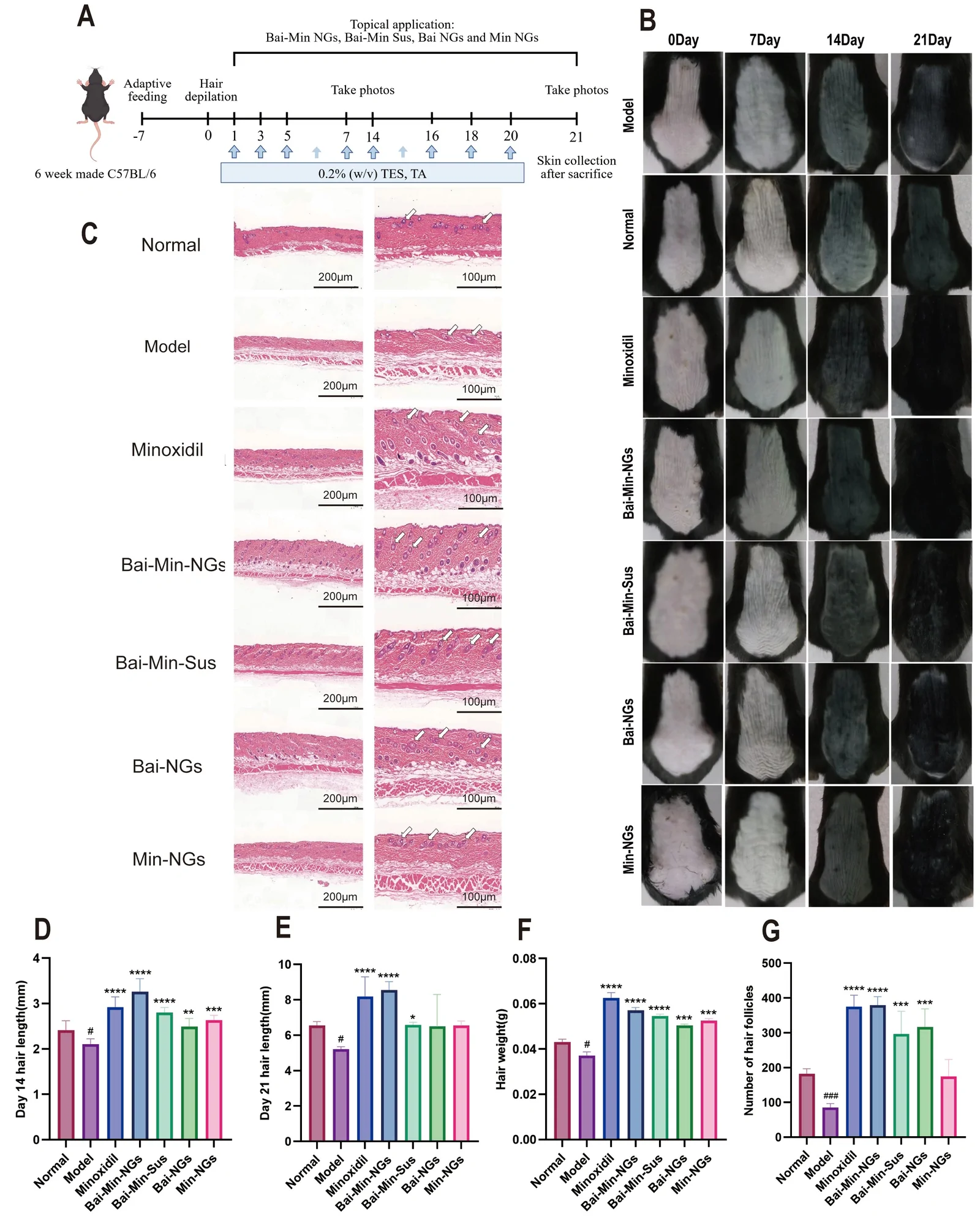
The treatment efficiency for AGA model mice. (**A**)The treatment schedule in C57BL/6 mice. (**B**) HE staining of the skin and (**C**) Photographs of mouse hair regrowth status. The length of regenerated hair at (**D**) 14th day and (**E**) 21st day. (**F**) The weight of regenerated hair at 21st. (**G**) The number of hair follicle at 21st day. (* *p* < 0.05, ** *p* < 0.01, *** *p* < 0.001, and **** *p* < 0.0001 (vs. model group); # *p* < 0.05 and ### *p* < 0.001 (vs. normal group)).

The hair length data on day 14 (Figure 4D) revealed a significant difference between the model and blank groups (*p* < 0.05), confirming the successful establishment of the alopecia model. At day 21, both hair length and hair weight (Figure 4E,F) in the positive control and Bai-Min-NGs groups differed significantly from those in the model group. These results demonstrate that both the positive drug and Bai-Min-NGs exert potent hair-growth-promoting effects.

#### 3.4.1. Macroscopic Observation of Hair Regeneration

As illustrated in Figure 4B, the Bai-Min-NGs group exhibited the most potent hair regrowth efficacy. After 7 days of treatment, the skin in both the Bai-Min-NGs and positive control groups was the first to transition to gray, indicating the entry of hair follicles into the anagen phase; conversely, no significant color change was observed in the model group. By day 14, the Bai-Min-NGs group achieved extensive pigmentation along with the emergence of nascent hair, demonstrating growth kinetics significantly superior to those of the single-drug groups. By day 21, the denuded area in this group was completely covered by high-density, dark-colored terminal hair, comparable to the positive control group, whereas the hair in the model group remained sparse and fine.

#### 3.4.2. Quantitative Analysis and Histological Evaluation of Hair Regeneration

Quantitative analysis (Figure 4D–F) confirmed that the length and weight of nascent hair in the Bai-Min-NGs group were significantly higher than those in the single-drug groups, demonstrating a clear synergistic effect.

Hematoxylin and eosin (H&E) staining further elucidated the underlying microscopic mechanism (Figure 4C). In the model group, most hair follicles remained in the telogen phase with shrunken structures; in contrast, the Bai-Min-NGs group exhibited a large number of hypertrophic anagen follicles penetrating deep into the dermis. Quantitative follicle counting (Figure 4G) revealed that the number of hair follicles in this group was significantly higher than in the single-drug groups and comparable to the levels observed in the positive control group. These results indicate that the composite formulation effectively activates telogen follicles, inducing and maintaining their transition into the anagen phase, thereby significantly promoting morphological hair regeneration.

### 3.5. Mechanisms of Reshaping the Hair Follicle Niche via Wnt/β-Catenin Pathway

The Wnt/β-catenin signaling pathway is a pivotal regulator of proliferation and differentiation across various tissues [33,34]. It is widely recognized in the field that this pathway plays a fundamental role in hair follicle morphogenesis, maintenance, and anagen induction. Furthermore, Wnt/β-catenin signaling is of paramount importance for the activation, proliferation, and differentiation of hair follicle stem cells (HFSCs) [35].

To further elucidate the synergistic mechanism between baicalin and minoxidil, quantitative reverse transcription-polymerase chain reaction (RT-PCR) was employed to determine the gene expression levels of growth factors associated with the progression of AGA. Concurrently, Western blot (WB) analysis was utilized to assess the expression of key proteins involved in the Wnt/β-catenin signaling pathway [19].

**Figure 5:**
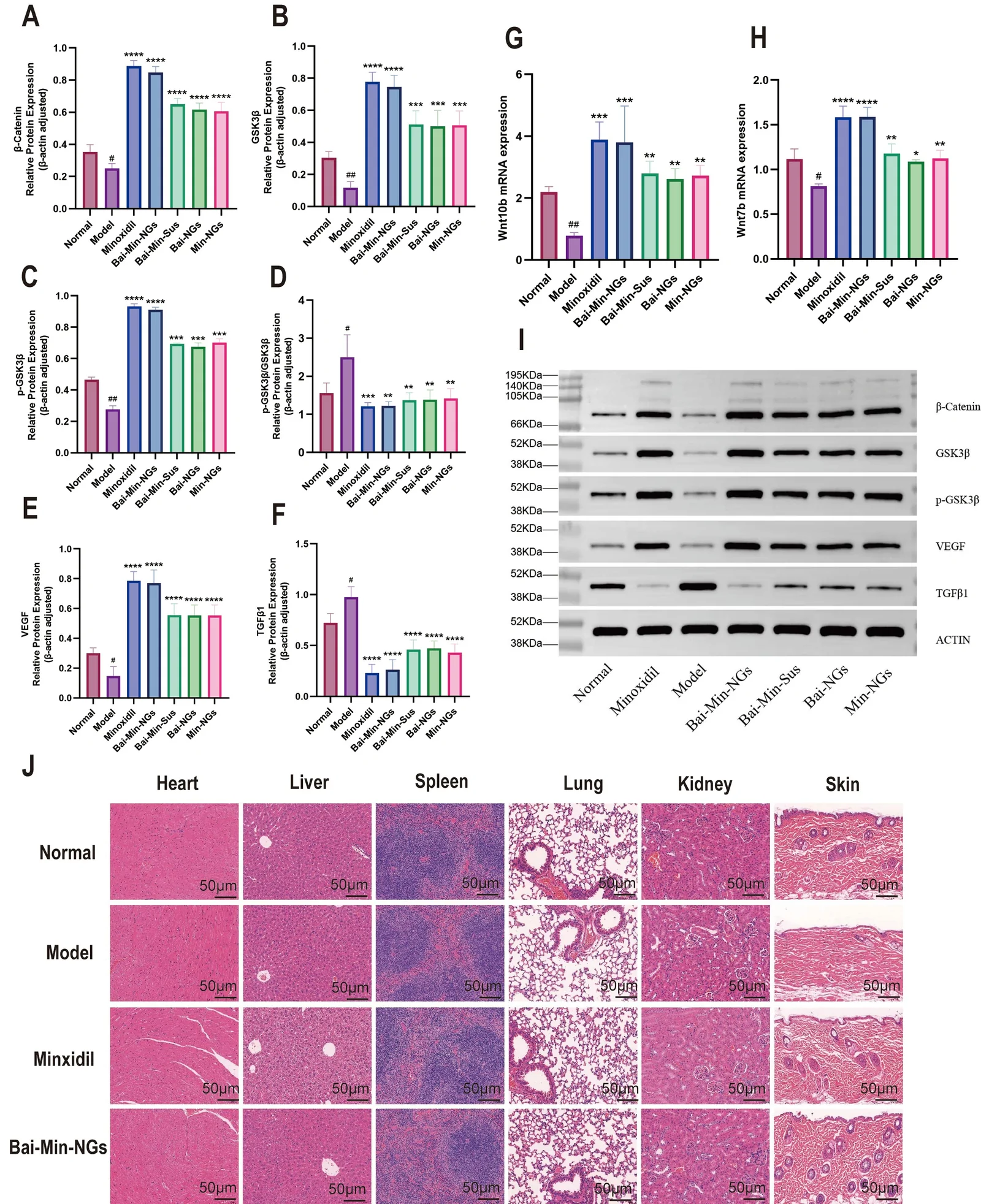
Regulation of the Wnt/β-catenin signaling pathway and safety evaluation in vivo. Relative protein expression levels of β-Catenin (**A**), GSK3β (**B**), p-GSK3β (**C**), the ratio of p-GSK3B/GSK3β (**D**), VEGF (**E**) and TGFβ1 (**F**) in skin tissues. The mRNA expression levels of Wnt (**G**) 10b and (**H**) Wnt 7b. (**I**) Relative proteins expression. (**J**) HE staining of major organs and skin in mice. (* *p* < 0.05, ** *p* < 0.01, *** *p* < 0.001, and **** *p* < 0.0001 (vs. model group); # *p* < 0.05 and ## *p* < 0.01 (vs. normal group)).

The results are presented in Figure 5. At the upstream level of the signaling pathway, qPCR analysis (Figure 5G,H) showed that the mRNA expression levels of *Wnt7b* and *Wnt10b* in the skin were significantly upregulated in all treatment groups compared to the model group. Among these, the minoxidil and Bai-Min-NGs groups exhibited the most pronounced increases (*Wnt7b*, *p* < 0.0001; *Wnt10b*, *p* < 0.001), indicating activation of the Wnt signaling pathway. Moving to the signal transduction level, WB quantification (Figure 5B–D) revealed that GSK3β protein expression was elevated in both the minoxidil and Bai-Min-NGs groups relative to the model group. Moreover, the levels of phosphorylated GSK3β (p-GSK3β) and the p-GSK3β/GSK3β ratio were significantly increased, suggesting inhibition of GSK3β activity. Consistent with these changes, the protein expression of β-catenin was markedly upregulated (Figure 5A), further confirming the activation of the Wnt/β-catenin signaling pathway. Representative protein bands are shown in Figure 5I.

With regard to pathway functional output (Figure 5E,F), the minoxidil and Bai-Min-NGs groups exhibited a significant upregulation of VEGF expression, along with a marked downregulation of TGF-β1, compared to the model group. These findings suggest that the treatment effectively optimized the local microenvironment to promote angiogenesis and support hair follicle growth. When compared intergroup, the minoxidil group showed a significantly greater upregulation of key molecular markers—including Wnt7b, Wnt10b, p-GSK3β, β-catenin, and VEGF—than the Bai-Min-NGs group (*p* < 0.05). Furthermore, In the 7-day repeated skin irritation test using New Zealand white rabbits, no obvious erythema, eschar or edema was observed in either the Bai-Min-NGs group or the normal saline control group at all observation time points. The daily skin irritation score for each animal in both groups was 0, with a mean cumulative score of 0. According to the Technical Specification for Cosmetic Safety (2015 Edition), Bai-Min-NGs were classified as non-irritating (Table 2). H&E staining (Figure 5J) revealed that the model group displayed compromised skin architecture, characterized by reduced hair follicle density and disorganized follicular arrangement. In contrast, following treatment with either minoxidil or Bai-Min-NGs, the skin structure was substantially restored, accompanied by a marked increase in the number of regularly arranged hair follicles. Importantly, no significant pathological damage was observed in major organs (heart, liver, spleen, lungs, and kidneys), demonstrating the favorable in vivo biosafety profile of both treatments.

## 4. Discussion

The prepared Bai-Min-NEs exhibited a uniform size distribution (droplet diameter < 200 nm) and stable negative zeta potential. Sub-micron particles are known to enhance skin penetration via intercellular and follicular routes, while surfactants within the nanoemulsion system disrupt stratum corneum lipid bilayers to improve transdermal delivery. Although the negative surface charge limits non-specific adsorption, it supports colloidal stability and particle diffusion within the skin [36]. TEM and rheological analysis confirmed the high dispersibility and structural integrity of Bai-Min-NEs in the hydrogel matrix. The storage modulus (G′) consistently exceeded the loss modulus (G″) across the tested range, indicating an elasticity-dominant microstructure that ensures physical stability and shear-thinning behavior favorable for topical application [37]. FT-IR spectra showed no irreversible drug-excipient interactions, preserving the bioactivity of Baicalin and Minoxidil.

Effective spatiotemporal distribution and prolonged retention are critical for local anti-alopecia therapy [38]. In vitro release kinetics demonstrated controlled-release characteristics of Bai-Min-NGs, extending drug residence in the cutaneous microenvironment. Specifically, the mathematical fitting confirmed a Super Case-II transport mechanism (*n* > 0.89), verifying that the swelling-controlled matrix relaxation guarantees constant zero-order release kinetics, thereby preventing rapid local drug depletion. Bai-Min-NGs significantly promoted the proliferation and migration of dermal papilla cells (DPCs). At the cellular level, quantitative flow cytometry (Figure 3E) and CLSM imaging (Figure 3F) confirmed that Bai-Min-NGs significantly enhanced cellular uptake and promoted intracellular lysosomal accumulation in DPCs. The observed strong colocalization with LysoTracker Red directly substantiates that the nanogel enters DPCs primarily via an active endo-lysosomal trafficking pathway. At the molecular level, the Wnt/β-catenin axis is essential for hair follicle cycling and stem cell activation [39,40,41]. qPCR revealed upregulated *Wnt7b* and *Wnt10b* transcripts in DPCs after treatment. Bai-Min-NGs also increased GSK3β phosphorylation, thereby inhibiting β-catenin degradation. This cascade triggered β-catenin nuclear translocation and activated hair growth-related gene transcription, explaining the telogen-to-anagen transition.

Although Bai-Min-NGs exhibit favorable therapeutic potential in the alopecia model, several limitations of the present study warrant further investigation. First, the colloidal stability of this nanogel during long-term storage and under physiologically relevant microenvironments has not been systematically evaluated. Longitudinal continuous monitoring of particle size, PDI, zeta potential and particle aggregation behavior is required to verify its shelf-life quality attributes. Second, although no obvious skin irritation was observed in the 7-day repeated topical application test with New Zealand white rabbits and no pathological alterations were detected in the histological evaluation of major organs from mice, its systemic safety, including hematological indices, serum biochemical parameters and local immune microenvironment responses following long-term repeated administration, remains to be further validated. Future studies should consolidate the biosafety evidence through systematic long-term toxicological assessments. Moreover, it is worthwhile to explore the possibility of combining this nanoplatform with physical stimuli such as low-level laser therapy (LLLT), so as to establish an integrated therapeutic strategy with chemo-biological and physical synergism and accelerate its clinical translation.

## 5. Conclusions

In summary, this study successfully developed a novel dual-drug-loaded nanogel delivery system: Bai-Min-NGs, providing a highly promising and innovative strategy to overcome the bottlenecks of poor transdermal permeability and low bioavailability associated with traditional topical treatments for AGA.

Leveraging the three-dimensional synergistic effect between the high-pressure homogenized nanoemulsion core and the gel network, Bai-Min-NGs effectively overcome the robust physical barrier of the stratum corneum. This system not only achieves prolonged retention and controlled release of Baicalin and Minoxidil within the deep layers of the skin but also targets DPCs via efficient endocytic pathways. At the cellular level, this high-efficiency intracellular drug accumulation significantly reverses hair follicle regression and markedly enhances the proliferation and directional migration activities of DPCs. More importantly, in-depth molecular mechanism studies revealed that the hair-growth-promoting efficacy of the formulation is closely associated with the cascade activation of the Wnt/β-catenin pathway, accompanied by the upregulation of its expression. This delivery platform not only significantly upregulates the expression of key upstream ligands (*Wnt7b* and *Wnt10b*) but also effectively blocks the proteasomal degradation pathway of β-catenin by inducing the inhibitory phosphorylation of GSK3β. This process promotes the substantial stabilization and intracellular enrichment of the core transcription factor β-catenin, thereby restarting the hair Anagen phase at both the genetic and protein levels.

In conclusion, as an intelligent nanotherapeutic platform integrating “enhanced transdermal penetration, targeted controlled release, and multi-target synergistic activation”, Bai-Min-NGs have not only demonstrated superior efficacy in reversing AGA across both in vitro and in vivo models but also clearly elucidated the underlying molecular pathways. These findings provide a robust theoretical foundation for the application of combination therapy strategies in the field of hair regeneration and open broad avenues for the clinical translation of next-generation transdermal drug delivery systems.

## Figures and Tables

**Table 1:** In vitro release kinetic parameters and model fitting of Baicalin and Minoxidil from Bai-Min-NGs.

Kinetic Models	Mathematical Equation	Active Drug	Fitted Equation	k
Zero -order	Q = k_0_·t + a	Min in NGs Bai in NGs	Q = 3.9713t − 4.7891 Q = 0.7751t − 0.6348	3.9713 %/h 0.7751 %/h
First-order model	ln(100 − Q) = −k_1_·t + a	Min in NGs Bai in NGs	ln(100 − Q) = −0.1011t + 4.8715 ln(100 − Q) = −0.0085t + 4.6138	0.1011 h^−1^ 0.0085 h^−1^
Higuchi model	Q = k_H_·t^1/2^ + a	Min in NGs Bai in NGs	Q = 19.7883t1/2 − 22.7026 Q = 3.8855t1/2 − 4.1824	19.7883 %/h^1/2^ 3.8855 %/h^1/2^
Korsmeyer-Peppas model (Mt/M∞ ≤ 0.60)	ln(Mt/M∞) = n·ln(t) + ln(k_KP_)	Min in NGs Bai in NGs	ln(Mt/M∞) = 1.2429ln(t) − 4.1131 ln(Mt/M∞) = 1.4553ln(t) − 4.2199	0.0164 h^−n^ 0.0147 h^−n^

**Table 2:** Skin irritation scores of New Zealand white rabbits after 7 consecutive days of repeated topical application of Bai-Min-NGs.

TIME (Day)	Bai-Min-NGs	Ctrl.
1	0	0
2	0	0
3	0	0
4	0	0
5	0	0
6	0	0
7	0	0
Mean score per animal per day	0	0

## Data Availability

The raw data generated and analyzed in this study are available from the corresponding author upon request.

## References

[1] Oiwoh S.O., Enitan A.O., Adegbosin O.T., Akinboro A.O., Onayemi E.O. (2024). Androgenetic Alopecia: A Review. Niger. Postgrad. Med. J..

[2] Ghonemy S., Alarawi A., Bessar H. (2021). Efficacy and safety of a new 10% topical minoxidil versus 5% topical minoxidil and placebo in the treatment of male androgenetic alopecia: A trichoscopic evaluation. J. Dermatol. Treat..

[3] Suchonwanit P., Thammarucha S., Leerunyakul K. (2019). Minoxidil and its use in hair disorders: A review. Drug Des. Devel. Ther..

[4] Asadi L., Mahmoudie T., Akbari M., Behbudi G. (2026). Biocompatible Titania for Drug Delivery and Tissue Engineering. Regen. Eng. Transl. Med..

[5] Chen L., Fan B., Gu H., Yang L., Li X. (2022). Effects of Baicalin on Alopecia and the Associated Mechanism. BioMed Res. Int..

[6] Rodríguez-Acosta G.L., Hernández-Montalbán C., Vega-Razo M.F.S., Castillo-Rodríguez I.O., Martínez-García M. (2021). Nanomedical Applications of Amphiphilic Dendrimeric Micelles. Curr. Med. Chem..

[7] Lei Y., Jiang W., Peng C., Wu D., Wu J., Xu Y., Yan H., Xia X. (2024). Advances in polymeric nano-delivery systems targeting hair follicles for the treatment of acne. Drug Deliv..

[8] Prow T.W., Grice J.E., Lin L.L., Faye R., Butler M., Becker W., Wurm E.M.T., Yoong C., Robertson T.A., Soyer H.P. (2011). Nanoparticles and microparticles for skin drug delivery. Adv. Drug Deliv. Rev..

[9] Sadighian S., Mahmoudie T., Kordloo A., Heydari M.R., Khanmohammadi A., Kaboli Z., Forouzideh N. (2026). Magnetic-Graphene-Oxide-Incorporated Alginate Hydrogel Beads as a Potential Controlled Drug Delivery System. Gels.

[10] Shen H., Qiao Y., Zhang C., Ma Y., Chen J., Cao Y., Zheng W. (2024). A matrix metalloproteinase-sensitive hydrogel combined with photothermal therapy for transdermal delivery of deferoxamine to accelerate diabetic pressure ulcer healing. Chin. Chem. Lett..

[11] Choi B.Y. (2020). Targeting Wnt/β-Catenin Pathway for Developing Therapies for Hair Loss. Int. J. Mol. Sci..

[12] Alhasso B., Ghori M.U., Rout S.P., Conway B.R. (2023). Development of a Nanoemulgel for the Topical Application of Mupirocin. Pharmaceutics.

[13] Siddiqui A., Jain P., Alex T.S., Ali M.A., Hassan N., Haneef J., Naseef P.P., Kuruniyan M.S., Mirza M.A., Iqbal Z. (2022). Investigation of a Minocycline-Loaded Nanoemulgel for the Treatment of Acne Rosacea. Pharmaceutics.

[14] Luo H., Wang X., Fang M., Yu H., Gui L., Wu Z., Sheng J., Li F. (2024). Combined Hydroxyethyl Starch Luteolin Nanocrystals for Effective Anti-Hyperuricemia Effect in Mice Model. Int. J. Nanomed..

[15] Asasutjarit R., Leenabanchong C., Theeramunkong S., Fristiohady A., Yimsoo T., Payuhakrit W., Sukatta U., Fuongfuchat A. (2023). Formulation optimization of sterilized xanthones-loaded nanoemulgels and evaluation of their wound healing activities. Int. J. Pharm..

[16] Hespeler D., Knoth D., Keck C.M., Müller R.H., Pyo S.M. (2019). smartPearls^®^ for dermal bioavailability enhancement—Long-term stabilization of suspensions by viscoelasticity. Int. J. Pharm..

[17] Calienni M.N., Martínez L.M., Izquierdo M.C., Alonso S.d.V., Montanari J. (2023). Rheological and Viscoelastic Analysis of Hybrid Formulations for Topical Application. Pharmaceutics.

[18] Dümichen A., Lucas H., Trutschel M.L., Mäder K. (2023). Development and Characterization of Novel In-Situ-Forming Oleogels. Pharmaceutics.

[19] He Z., Zhang Y., Liu Z., Guo T., Ai X., He Y., Hou X., Feng N. (2024). Synergistic treatment of androgenetic alopecia with follicular co-delivery of minoxidil and cedrol in metal–organic frameworks stabilized by covalently cross-linked cyclodextrins. Int. J. Pharm..

[20] Yan A., Ruan R., Zhu X., Qiang W., Guan Y., Yu Q., Sun H., Liu M., Zhu H. (2023). Co-delivery of minoxidil and tocopherol acetate ethosomes to reshape the hair Follicular Microenvironment and promote hair regeneration in androgenetic alopecia. Int. J. Pharm..

[21] Fan X., Chen J., Zhang Y., Wang S., Zhong W., Yuan H., Wu X., Wang C., Zheng Y., Wei Y. (2022). Alpinetin promotes hair regeneration via activating hair follicle stem cells. Chin. Med..

[22] Zhang J., Shu J., Stout R.W., Russo P.S., Liu Z. (2024). Solubilization of Paclitaxel with Natural Compound Rubusoside toward Improving Oral Bioavailability in a Rodent Model. Pharmaceutics.

[23] Bagul U.S., Khot S.V., Ashtekar K.S., Monde A.A., Kolhe O.H., Tagalpallewar A.A., Kokare C.R. (2024). Fabrication of acetazolamide loaded leciplex for intraocular delivery: Optimization by 3(2) full factorial design, in vitro, ex vivo and in vivo pharmacodynamics. Int. J. Pharm..

[24] Li Y., Wang J., Vora L.K., Sabri A.H.B., McGuckin M.B., Paredes A.J., Donnelly R.F. (2024). Dissolving microarray patches loaded with a rotigotine nanosuspension: A potential alternative to Neupro^®^ patch. J. Control. Release.

[25] Wang X., Liu X., Guo Y., Gong T., Lu W., Han M., Guo Y., Wang X. (2024). The Remarkable Anti-Breast Cancer Efficacy and Anti-Metastasis by Multifunctional Nanoparticles Co-Loading Squamocin, R848 and IR 780. Int. J. Nanomed..

[26] El-Feky G.S., El-Banna S.T., El-Bahy G.S., Abdelrazek E.M., Kamal M. (2017). Alginate coated chitosan nanogel for the controlled topical delivery of Silver sulfadiazine. Carbohydr. Polym..

[27] Batheja P., Sheihet L., Kohn J., Singer A.J., Michniak-Kohn B. (2011). Topical drug delivery by a polymeric nanosphere gel: Formulation optimization and in vitro and in vivo skin distribution studies. J. Control. Release.

[28] Karnam S., Jindal A.B., Paul A.T. (2024). Quality by design-based optimization of teriflunomide and quercetin combinational topical transferosomes for the treatment of rheumatoid arthritis. Int. J. Pharm..

[29] Grivet-Brancot A., Buscemi M., Ciardelli G., Bronco S., Sartori S., Cassino C., Al Kayal T., Losi P., Soldani G., Boffito M. (2024). Cord Blood Platelet Lysate-Loaded Thermo-Sensitive Hydrogels for Potential Treatment of Chronic Skin Wounds. Pharmaceutics.

[30] Wang J., Risola D.D., Mattioli R., Zoratto N., Mosca L., Meo C.D., Matricardi P. (2025). Hyaluronan-Cholesterol nanogels embedding betamethasone for the treatment of skin inflammatory conditions. Int. J. Pharm..

[31] Abid F., Virgo E., Kennewell T.L., Khetan R., Haidari H., Kopecki Z., Song Y., Garg S. (2024). The Acid-Buffered Engineered Gel Promotes In Vitro Cutaneous Healing and Fights Resistant Bacteria in Wounds. Pharmaceutics.

[32] Mi X., Hu M., Dong M., Yang Z., Zhan X., Chang X., Lu J., Chen X. (2021). Folic Acid Decorated Zeolitic Imidazolate Framework (ZIF-8) Loaded with Baicalin as a Nano-Drug Delivery System for Breast Cancer Therapy. Int. J. Nanomed..

[33] Devjani S., Ezemma O., Kelley K.J., Stratton E., Senna M. (2023). Androgenetic Alopecia: Therapy Update. Drugs.

[34] Yuan L., Pan M., Deng H., Yang T., Liu Q., Chen W., Li X., Liu J., Xu K., He X. (2025). Enhanced hair follicle accumulation and androgenetic alopecia treatment of finasteride by topical nanoparticles hydrogel system. Chin. Chem. Lett..

[35] Ryu Y.C., Kim Y.R., Park J., Choi S., Ryu W.J., Kim G.U., Kim E., Hwang Y., Kim H., Han G. (2022). Pyruvate Kinase M2 Promotes Hair Regeneration by Connecting Metabolic and Wnt/β-Catenin Signaling. Pharmaceutics.

[36] Nastiti C.M.R.R., Ponto T., Abd E., Grice J.E., Benson H.A.E., Roberts M.S. (2017). Topical Nano and Microemulsions for Skin Delivery. Pharmaceutics.

[37] Al-Barghouthy E.Y., Hamed S., Mehyar G.F., AlKhatib H.S. (2025). Comparative Evaluation of Spreadability Measurement Methods for Topical Semisolid Formulations/A Scoping Review. Gels.

[38] Gu Y., Bian Q., Zhou Y., Huang Q., Gao J. (2022). Hair follicle-targeting drug delivery strategies for the management of hair follicle-associated disorders. Asian J. Pharm. Sci..

[39] Wen L., Fan Z., Huang W., Miao Y., Zhang J., Liu B., Zhu D., Dai D., Zhang J., Le D. (2024). Retinoic acid drives hair follicle stem cell activation via Wnt/β-catenin signalling in androgenetic alopecia. J. Eur. Acad. Dermatol. Venereol. JEADV.

[40] Mayumi I., Kenji K., Masahiko T., Masaaki M. (2002). Label-retaining cells in the bulge region are directed to cell death after plucking, followed by healing from the surviving hair germ. J. Investig. Dermatol..

[41] Stenn S.K., Paus R. (2001). Controls of hair follicle cycling. Physiol. Rev..

